# Open-source spring-driven syringe pump with 3D-printed components for microfluidic applications

**DOI:** 10.1016/j.ohx.2024.e00550

**Published:** 2024-07-06

**Authors:** Se Been Park, Joong Ho Shin

**Affiliations:** a Industry 4.0 Convergence Bionics Engineering, Pukyong National University, Republic of Korea; b Major of Biomedical Engineering, Division of Smart Healthcare, College of Information Technology and Convergence, Pukyong National University, Busan 48513, Republic of Korea

**Keywords:** Microfluidics, Syringe pump, 3D printing, Portable, Nonelectric

## Abstract

The operation of microfluidic devices requires precise and constant fluid flow. Microfluidic systems in low-resource settings require a portable, inexpensive, and electricity-free pumping approach due to the rising demand for microfluidics in point-of-care testing (POCT). Open-source alternatives, employing 3D printing and motors, offer affordability. However, using motors require electrical power, which often relies on external sources, hindering the on-site use of open-source pumps. This study introduces a spring-driven, 3D-printed syringe pump, eliminating the need for an external power source. The syringe pump is operated by the flat spiral spring’s torque. By manually winding up the mainspring, the syringe pump can be operated without electricity. Various flow rates can be achieved by utilizing different syringe sizes and choosing the right gear combinations. All the parts of the syringe pump can be fabricated by 3D printing, requiring no additional components that require electricity. It operates by winding a mainspring and is user-friendly, allowing flow rate adjustments by assembling gears that modulate syringe plunger pushing velocity. The fabrication cost is $25–30 and can be assembled easily by following the instructions. We expect that the proposed syringe pump will enable the utilization of microfluidic technologies in resource-limited settings, promoting the adoption of microfluidics. Detailed information and results are available in the original research paper (https://doi.org/10.1016/j.snb.2024.135289).

## Graphical abstract

**Figure:**
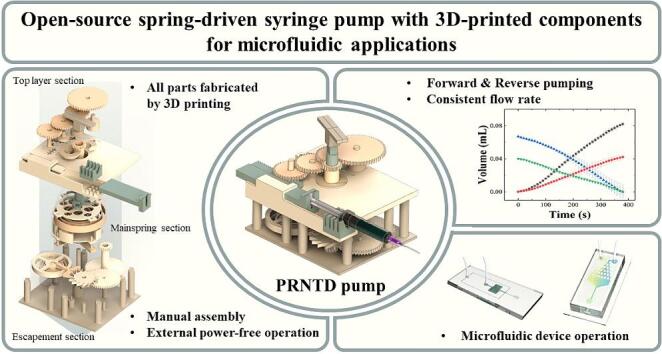

## 1 Hardware in context

Microfluidics is a technology that manipulates small quantities of fluids in the range of 10^−9^ to 10^−18^ L through channels with dimensions ranging from tens to hundreds of micrometers [1], [2]. Microfluidics offers advantages by allowing for analysis and experimentation with small fluid quantities, resulting in decreased expenses when utilizing costly experimental and analytical substances. Microfluidics is utilized in a variety of fields, including analyses such as electrochemical sensing [3] and environmental monitoring [4], the generation of droplets for droplet digital polymerase chain reaction (ddPCR) [5], drug screening [6], [7], and cell screening [8], as well as in cell culture [9] and organ-on-a-chip system [10], DNA analysis [11], [12], and fuel cells [13]. Microfluidics is widely used in a variety of fields; however, the operation of a microfluidic device requires a syringe pump.

A syringe pump can cost between $350 [14] to approximately $3,500 [15] depending on the functions and specifications of the pump. The cost of conventional syringe pump can be a burden not only to labs in resources limited settings, but also labs in developed countries. To mitigate this, many efforts have been made to develop open-source syringe pumps, allowing users to build and customize syringe pumps at a minimal cost. By providing CAD file, component specs, and the code file online, users can build their own syringe pump with cost as little as $75 [16] to $400 [17]. A standard form of an open-source syringe pump consists of a stepper motor, motor controller, lead screw and a frame that allows back and forth movement of a pusher block which pushes the syringe plunger of a plastic or glass syringe. One of the earlier open-source syringe pump was developed by Wijnen et al [18], demonstrating a construction of a functional pump using only open-source software, open-source 3D printer and open-source computer (Raspberry Pi). It is worth noting that this pump has been modified in different variations for different purposes. For example, a PCB for the pump controller was added to deliver a bolus of fluid across the user-specified timing for neuroscience research [19], and multiple pumps are connected to an open-source microfluidic control instrument to generate monodisperse emulsion [17].

One of the advantages of developing and using open-source syringe pumps is that modifications and upgrades using open-source computers such as Arduino and Raspberry Pi allow the pump to work as a stand-alone device that does not depend on a PC [20], imbues the pump with additional features such as wireless connectivity [21] and perform specific tasks by programming. Arduino board has been widely used for many applications such as programming the pump to deliver drugs and contrast agents for in-vivo imaging [22]; granting a precise feedback-control by integrating with a pressure sensor [23]; and being used as a programmable learning tool for developing a multichannel syringe pump [24]. Furthermore, Raspberry Pi has been used to control two pumps simultaneously to generate cell lysates [25], and operating microfluidic devices for single cell sequencing [17], [26].

Despite the low cost, high versatility and high expandability, constructing an open-source syringe pump can be a difficult task especially when there are many parts to buy and they have to be obtained from multiple vendors. One way to eliminate such a difficulty is to use the parts that are included in a commercial product. Baas et al. report an assembly of a programmable syringe pump using mostly the parts included in Ender3 3D printer kit. By using the parts provided in the kit, a set of three syringe pumps can be built [27]. Recently, our research group developed a non-electric syringe pump that can be fabricated using the clockwork mechanism retrieved from a commercial cooking timer [28]. However, the syringe pump requires specific model of commercial cooking timer to fabricate, which limits its replication.

Another way to eliminate the difficulty of gathering parts is to build a syringe pump using only a 3D printed parts. Therefore, our group recently developed a nonelectric syringe pump that can be assembled by using only 3D printed parts [29]. The syringe pump was named precise, rapid-prototyped, nonelectric, torque-driven (PRNTD) pump. Unlike commercially available syringe pumps and existing open-source syringe pumps, the PRNTD pump consists only of 3D printed parts, which eliminates the need to gather commercial parts such as motors, screws, bolts and nuts that are required for the assembly. The pump operates without any electrical power, instead, it winds a flat spiral spring and is operated by clockwork mechanism to push or pull the syringe plunger for fluid delivery. Flow rate adjustment is achieved by using a specific combination of gears and syringe sizes. The PRNTD pump is, to the best of our knowledge, a first functional lab instrument that is not run by electricity and is assembled by using nothing but 3D printed parts.

Our previously published work reports the performance of the PRNTD pump and demonstrates useful and important microfluidic applications such as gradient generation and droplet generation. Although the paper also provides the DOI address from which STL files can be downloaded, additional instruction for assembling the pump can benefit its potential users. Thus, in this paper, we present a detailed step-by-step instruction on how to assemble the PRNTD pump. The fabrication cost of the syringe pump is $25–30, calculated based on the consumer price of the PLA filament used in this study.

## 2 Hardware description

### 2.1 Overview of 3D printed syringe pump

The PRNTD pump is made up of three main sections: the mainspring, the escapement, and the top layer. These sections are interconnected, with the mainspring providing torque as the pump's power source, the escapement regulating the torque from the mainspring, and the top layer converting the torque transmitted by the mainspring into linear motion by pushing the syringe plunger.

All components of the pump can be fabricated by 3D printing, which allows anyone that have basic knowledge about 3D printing to build one. After 3D printing, the pump is ready to use once all components are assembled according to the assembly instructions. The PRNTD pump comprised 27 parts and dimensions are 13.9 × 12.7 × 12.8 cm. The total time required for 3D printing all parts is 4 days, 4 h, completed over four separate runs.

Being able to build a pump using only 3D printed parts have several advantages. Firstly, users do not have to gather the parts required for assembly. Secondly, because it does not use any electronic parts, the cost is very cheap and in fact it is by far the cheapest open-source syringe pump reported [16]. Additionally, the pump does not rely on electricity, enhancing its portability and accessibility for various on-site applications, whereas the majority of open-source pumps require an external power source for operation.

The spring-based pump however does have several limitations and disadvantages. The inner workings of the pump involve many freely rotating parts, which require a delicate optimization of the 3D printer used. The optimization should ensure that the gears are not tightly fitted to their posts to minimize friction. However, if the gaps between the rotating parts are too large, the gears may not mesh properly, leading to unwanted backlash. Another disadvantage of the PRNTD pump is the limited controllability of the flow rate. Unlike the open-source syringe pumps that have control panels for digital flow rate control [20], [25], the PRNTD pump relies on the analog combination of gear ratio and the syringe size for setting the flow rate. This can be a daunting task when several different flow rates are required in an experiment. The analog flow rate setting also limits users from changing the flow rate in the middle of the pump’s operation, which is a feature that is often used in microfluidic experiments. Thus, the PRNTD pump may not be suitable for experiments requiring the screening of many flow rates are required, but it is ideal for applications that require pumping of liquid with a specified flow rate. Lastly, because the spiral spring is 3D printed, its torque is less than that provided by a metal spring or electrically driven stepper motors. Thus, the PRNTD pump’s flow rate can be affected by the back pressure, or the resistance of the system to which fluid is delivered. This results in large percent error of the flow rate, which can be as high as 27.8 % [29] However, the accuracy ideally should be in a single-digit value, preferably within ± 5 % [18]. In future research, PRNTD pump can be redesigned to use a threaded screw, limiting the amount of torque delivered to the spring from the back pressure, thereby improving accuracy.

### 2.2 PRNTD pump

• Researchers can build the syringe pump to operate the microfluidic devices that require consistent fluid flow.
• Researchers can build the syringe pump only with 3D printing and fabricate it for $25–30.
• The syringe pump operates without electricity using a 3D printed spiral spring, eliminating the need for additional components like motors and microprocessors.
• The syringe pump is lightweight, portable and can be used in low-resource settings with limited power supplies.

### 2.3 Design **files**

**Mainspring section.** The Mainspring section corresponds to the power supply of a commercial syringe pump. In the PRNTD pump, unlike a commercial syringe pump that depend on electricity to run a motor, the torque from the mainspring acts as the driving force. By rotating the handle of the PRNTD pump clockwise, the mainspring is wound up, and the torque generated as the mainspring unwinds serves as the power source for the syringe pump. The Mainspring section consists of the mainspring, spring barrel holder, spring barrel bottom, and spring barrel top (Table 1).

**Table 1:** Label, part name, and description for the components of the mainspring section.

Label	Part name	Description
M1	Spring barrel holder	
M2	Spring barrel bottom	
M3	Mainspring	
M4	Spring barrel top	

**Escapement section.** The Escapement section operates by utilizing the torque generated as the mainspring unwinds. This torque, transmitted through the torque reduction gear, rotates the escapement wheel, causing the pallet fork to oscillate. The pallet fork, in turn, imparts a pendulum-like motion to the balance wheel, ensuring a consistent unwinding speed of the mainspring. The Escapement section is comprised of the escapement frame, main gear, ratchet, torque reduction gear 1 and 2, escapement wheel, hairspring, pallet fork, and balance wheel (Table 2).

**Table 2:** Label, part name, and description for the components of the escapement section.

Label	Part name	Description
E1	Escapement frame	
E2	Main gear	
E3	Ratchet	
E4-1, E4-2	Torque reduction gear	
E5	Escapement wheel	
E6	Pallet fork	
E7	Balance wheel	
E8	Hairspring	

**Top layer section.** The top layer plays a role in adjusting the forward or backward speed of the pusher block in a commercial syringe pump. The torque from the mainspring is transmitted to the rack gear through the arrangement of gears, converting it into linear motion. The forward speed of the rack gear is adjusted to push the syringe plunger by controlling the gear ratio. The forward speed of the rack gear can be controlled by changing the gear ratio, accomplished by combining a total of four gears. In the syringe pump, different-sized syringes can be assembled by mounting a syringe holder. This includes 1-, 3-, or 5-mL disposable syringes, as well as 50-, 100-, 250-, or 500 µL Hamilton gastight glass syringes. Depending on the desired flow rate, different sizes of syringes can be mounted. The top layer section consists of a top layer frame, two main gear holding frames, four gear posts, a syringe holder, and a winding key in both forward pumping mode and reverse pumping mode. It is important to note that a forward pumping mode requires four velocity reduction gears, whereas a reverse pumping mode requires five velocity reduction gears (Table 3). To change the forward speed of the syringe plunger for flow rate adjustment, user can alter the positions of the gear posts and then assemble the velocity reduction gears of different numbers of gear teeth. Lastly, one of the six rack gears is selected according to the target gear ratio and syringe type. A disposable syringe requires mounting of rack gear R4-D1, R4-D2, or R4-D3 and syringe holder R6-P, whereas a glass syringe requires mounting of rack gear R4-G1, R4-G2, or R4-G3 and syringe holder R6-G. The detailed instructions explaining the correct combination of the velocity reduction gears and rack gears is shown in Table 7.

**Table 3:** Label, part name, and description for the components of the top layer section.

Label	Part name	Description
R1	Top layer frame	
R2-1, R2-2	main gear holding frame	
R3-1, R3-2, R3-3, R3-4, R3-5	Gear post	
R4-D1, R4-D2, R4-D3, R4-G1, R4-G2, R4-G3	Rack gear	
R5-60, R5-48, R5-36, R5-24, R5-12	Velocity reduction gear	
R6-D, R6-G	Syringe holder	
R7	Winding key	

## 3 Design files summary

The design files (Table 4) are available at https://doi.org/10.17632/28dxsg7x2t.1.

**Table 4:** Design files summary.

Design file name	File type	Open-source license	Location of the file	3D printing support requirement
Mainspring (M1)	CAD(.stl)	TAPR OHL	https://doi.org/10.17632/28dxsg7x2t.1	X
Spring barrel holder (M2)	CAD(.stl)	TAPR OHL	https://doi.org/10.17632/28dxsg7x2t.1	X
Spring barrel bottom (M3)	CAD(.stl)	TAPR OHL	https://doi.org/10.17632/28dxsg7x2t.1	X
Spring barrel top (M4)	CAD(.stl)	TAPR OHL	https://doi.org/10.17632/28dxsg7x2t.1	X
Escapement frame (E1)	CAD(.stl)	TAPR OHL	https://doi.org/10.17632/28dxsg7x2t.1	X
Main gear (E2)	CAD(.stl)	TAPR OHL	https://doi.org/10.17632/28dxsg7x2t.1	O
Ratchet (E3)	CAD(.stl)	TAPR OHL	https://doi.org/10.17632/28dxsg7x2t.1	O
Torque reduction gear (E4-1)	CAD(.stl)	TAPR OHL	https://doi.org/10.17632/28dxsg7x2t.1	X
Torque reduction gear (E4-2)	CAD(.stl)	TAPR OHL	https://doi.org/10.17632/28dxsg7x2t.1	X
Escapement wheel (E5)	CAD(.stl)	TAPR OHL	https://doi.org/10.17632/28dxsg7x2t.1	X
Pallet fork (E6)	CAD(.stl)	TAPR OHL	https://doi.org/10.17632/28dxsg7x2t.1	X
Balance wheel (E7)	CAD(.stl)	TAPR OHL	https://doi.org/10.17632/28dxsg7x2t.1	O
Hairspring (E8)	CAD(.stl)	TAPR OHL	https://doi.org/10.17632/28dxsg7x2t.1	X
Top layer frame (R1)	CAD(.stl)	TAPR OHL	https://doi.org/10.17632/28dxsg7x2t.1	O
Main gear holding frame (R2-1)	CAD(.stl)	TAPR OHL	https://doi.org/10.17632/28dxsg7x2t.1	X
Main gear holding frame (R2-2)	CAD(.stl)	TAPR OHL	https://doi.org/10.17632/28dxsg7x2t.1	X
Gear post (R3-1)	CAD(.stl)	TAPR OHL	https://doi.org/10.17632/28dxsg7x2t.1	O
Gear post (R3-2)	CAD(.stl)	TAPR OHL	https://doi.org/10.17632/28dxsg7x2t.1	O
Gear post (R3-3)	CAD(.stl)	TAPR OHL	https://doi.org/10.17632/28dxsg7x2t.1	O
Gear post (R3-4)	CAD(.stl)	TAPR OHL	https://doi.org/10.17632/28dxsg7x2t.1	O
Gear post (R3-5)	CAD(.stl)	TAPR OHL	https://doi.org/10.17632/28dxsg7x2t.1	O
Rack gear (R4-D1)	CAD(.stl)	TAPR OHL	https://doi.org/10.17632/28dxsg7x2t.1	O
Rack gear (R4-D2)	CAD(.stl)	TAPR OHL	https://doi.org/10.17632/28dxsg7x2t.1	O
Rack gear (R4-D3)	CAD(.stl)	TAPR OHL	https://doi.org/10.17632/28dxsg7x2t.1	O
Rack gear (R4-G1)	CAD(.stl)	TAPR OHL	https://doi.org/10.17632/28dxsg7x2t.1	O
Rack gear (R4-G2)	CAD(.stl)	TAPR OHL	https://doi.org/10.17632/28dxsg7x2t.1	O
Rack gear (R4-G3)	CAD(.stl)	TAPR OHL	https://doi.org/10.17632/28dxsg7x2t.1	O
Velocity reduction gear (R5_12)	CAD(.stl)	TAPR OHL	https://doi.org/10.17632/28dxsg7x2t.1	O
Velocity reduction gear (R5_24)	CAD(.stl)	TAPR OHL	https://doi.org/10.17632/28dxsg7x2t.1	X
Velocity reduction gear (R5_36)	CAD(.stl)	TAPR OHL	https://doi.org/10.17632/28dxsg7x2t.1	X
Velocity reduction gear (R5_48)	CAD(.stl)	TAPR OHL	https://doi.org/10.17632/28dxsg7x2t.1	X
Velocity reduction gear (R5_60)	CAD(.stl)	TAPR OHL	https://doi.org/10.17632/28dxsg7x2t.1	X
Syringe holder (R6-D)	CAD(.stl)	TAPR OHL	https://doi.org/10.17632/28dxsg7x2t.1	X
Syringe holder (R6-G)	CAD(.stl)	TAPR OHL	https://doi.org/10.17632/28dxsg7x2t.1	X
Winding key (R7)	CAD(.stl)	TAPR OHL	https://doi.org/10.17632/28dxsg7x2t.1	X

## 4 Bill of materials summary

For the fabrication of all components of the syringe pump, it requires 45.28 m (358 g) of PLA, including support for the printed objects. The total cost of building a syringe pump is $25–30 (Table 5).

**Table 5:** Bill of materials summary.

Designator	Component	Number	Cost perunit ($)	Total cost ($)	Source of materials	Material type
Syringe pump	All	0.48	$49.95	$25–30	https://ultimaker.com/materials/s-series-pla/	PLA

## 5 Build instructions

### 5.1 Printing **settings**

The quality and characteristics of 3D-printed objects, such as the PRNTD pump, depend on factors like the resolution of the 3D printer, material properties used in 3D printing, the minimum achievable feature size determined by slicing conditions, and the accuracy and resolution of the 3D-printed parts. Table 6 shows the 3D printer setting values used by the authors for the fabrication of the PRNTD pump. These 3D printing settings are also presented at supplementary files of previously published research [29]. The software Ultimaker Cura slicer (Ultimaker, Netherlands) was used for slicing and setting the print. Ultimaker S3 (Ultimaker, Netherlands) was used to build PRNTD pump in this study.

**Table 6:** 3D printing setting of the syringe pump.

Parameters	Values
Nozzle size	0.4 mm
Z-layer height	0.15 mm
Initial layer height	0.2 mm
Single-line and wall width	0.4 mm
Initial layer line width	120 %
wall thickness	0.8 mm
wall line count	2
top/bottom thickness	1 mm
top bottom layers	7
infill density	20 %
infill line distance	6 mm
infill pattern	triangle
infill line multiplier	1 mm
infill overlap percentage	0 %
infill layer thickness	0.15 mm
gradual infill steps	0 %
printing temperature	200 ℃
printing temperature initial layer	200 ℃
initial printing temperature	190 ℃
final printing temperature	185 ℃
build plate temperature	60 ℃
build plate temperature initial layer	60 ℃
print speed	70 mm/s
infill speed	70 mm/s
wall speed	45 mm/s
outer wall speed	23 mm/s
inner wall speed	45 mm/s
top/bottom speed	35 mm/s
travel speed	150 mm/s
initial layer speed	10 mm/s
retraction distance	6.5 mm
retraction speed	45 mm/s
travel avoid distance	3.0 mm
z hop height	2.0 mm

**Table 7:** Directions for assembling velocity reduction gear posts and velocity reduction gears.

Gear ratio	Scheme showing the insert locations of velocity reduction gear post and velocity reduction gear	Corresponding position number of velocity reduction gears and rack gear	
1: 81		Position 1	R5-60
		Position 2	R5-36
		Position 3	R5-36
		Position 4	R5-36
		Position 5	R4-D1 or R4-G1
1:90		Position 1	R5-60
		Position 2	R5-36
		Position 3	R5-24
		Position 4	R5-60
		Position 5	R4-D3 or R4-G3
1:108		Position 1	R5-60
		Position 2	R5-36
		Position 3	R5-36
		Position 4	R5-48
		Position 5	R4-D2 or R4-G2
1:120		Position 1	R5-60
		Position 2	R5-24
		Position 3	R5-36
		Position 4	R5-48
		Position 5	R4-D2 or R4-G2
1:135		Position 1	R5-60
		Position 2	R5-36
		Position 3	R5-36
		Position 4	R5-60
		Position 5	R4-D3 or R4-G3
1:144		Position 1	R5-60
		Position 2	R5-36
		Position 3	R5-48
		Position 4	R5-48
		Position 5	R4-D2 or R4-G2
1:180		Position 1	R5-60
		Position 2	R5-36
		Position 3	R5-48
		Position 4	R5-60
		Position 5	R4-D3 or R4-G3
1:192		Position 1	R5-60
		Position 2	R5-48
		Position 3	R5-48
		Position 4	R5-48
		Position 5	R4-D2 or R4-G2
1:240		Position 1	R5-60
		Position 2	R5-48
		Position 3	R5-48
		Position 4	R5-60
		Position 5	R4-D3 or R4-G3

### 5.2 Mainspring **assembly**

**Fig. 1:**
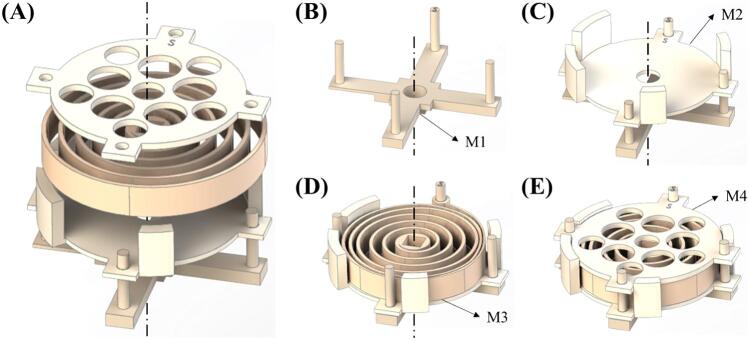
(A) Showing the exploded view of the mainspring section. (B), (C), (D), (E) Schematic showing the order of assembling mainspring section.
2) Slightly wind the mainspring (M3) to reduce its outer diameter, and while preventing the mainspring from unwinding, place M3 into M2 by assembling the looped end of the M3 with one of the prongs of M1. Make sure to insert the prong that is marked with ‘S’ into the looped end of M1 as shown in Fig. 1D.
3) Position spring barrel top (M4) by aligning the 'S' marked hole with the prong marked 'S', then finish the assembly as shown in Fig. 1E.

1) Assemble spring barrel bottom (M2) onto the spring barrel holder (M1) in a cross-shaped configuration (Fig. 1A). Align the post on M2 (Fig. 1B) with the hole next to 'S' marked on M1 (Fig. 1C).

### 5.3 **Escapement** assembly

**Fig. 2:**
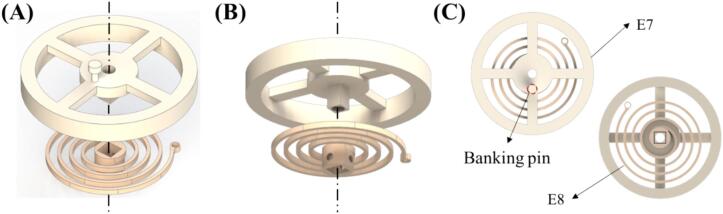
(A), (B) Showing the exploded view of the balance wheel and hairspring assembly. (C) Schematic showing the upside and downside of the assembled balance wheel and hairspring.
2) Assemble two torque reduction gears (E4-1 and E4-2), and escapement wheel (E5) onto escapement frame (E1) in sequence (Fig. 3A, Fig. 3B and Fig. 3C).

1) Assemble balance wheel (E7) and hairspring (E8) (Fig. 2A and Fig. 2B). Assemble the E7 and E8 according to Fig. 2C, ensuring that the rectangular post of E7 fits into the rectangular gap of E8.

**Fig. 3:**
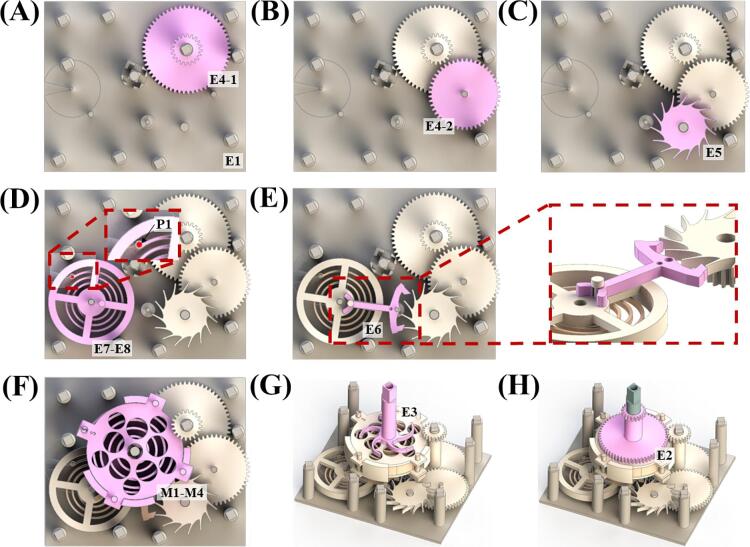
Schematics showing the escapement section assembly orders.
3) Assemble the assembled E7 and E8 onto E1 (Fig. 3D). Be cautious since E1′s posts are thin; assemble the hole at the end of E8′s strip onto E1′s post (P1) marked on Fig. 3D cautiously.
4) Position pallet fork (E6) with the surface labeled 'U' facing upwards. Assemble E6 onto E1 according to Fig. 3E. Ensure that E6 and banking pin of E7 engage together.
5) Assemble the assembled mainspring section onto the longest post of E1 (Fig. 3F). Ensure that the post marked 'S' aligns between the three posts on E1. Make sure that the elevation of the smaller gear of E4-1 is above the assembled complex of M1-M4.
6) Assemble ratchet (E3) onto the longest post of E1 (Fig. 3G). Adjust the direction of E3 to fit into the rectangular gap of M1.
7) Assemble main gear (E2) onto E3, make sure that E3′s gear fits inside the internal gear of E2. Also ensure that E2 and the small gear of E4-1 mesh together (Fig. 3H).

### 5.4 Top layer section assembly

**Fig. 4:**
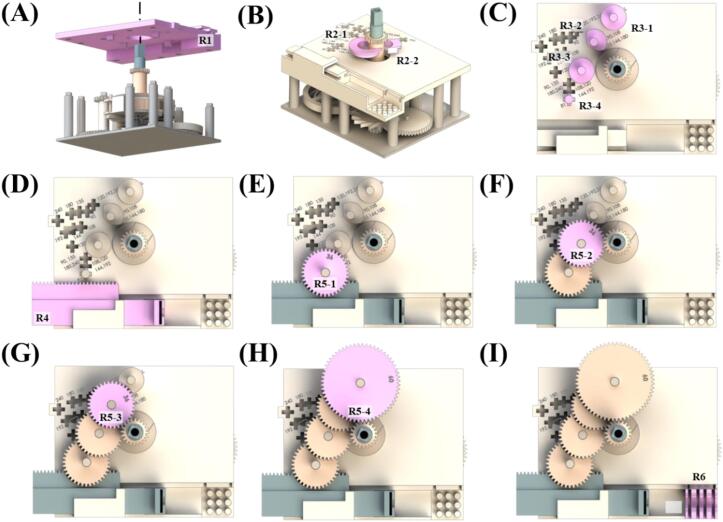
Schematics showing the top layer section assembly orders of forward pumping.
2) Insert main gear holding frames (R2-1 and R2-2) between R1 and E2 to fill the gap (Fig. 4B).
3) Assemble gear posts (R3-1, R3-2, R3-3, R3-4) onto R1 at positions corresponding to the target gear ratio (Fig. 4C). Check the numbers marked on the R1 and assemble the posts according to the desired gear ratio (Table 7).
4) Assemble rack gear (R4) according to the desired gear ratio (Table 7). When using a disposable syringe, use R4-D1, R4-D2, or R4-D3. When using a glass syringe, use R4-G1, R4-G2, or R4-G3 (Fig. 4D).
5) Assemble R5-1 (Fig. 4E), R5-2 (Fig. 4F), R5-3 (Fig. 4G), and R5-4 (Fig. 4H) in sequence.
6) Assemble syringe holder (R6). When using a disposable syringe, use R6-D. When using a glass syringe, use R6-G (Fig. 4I).

1) Assemble top layer section frame (R1) onto E1. Verify the positions of E1′s posts and the holes in R1 before assembly (Fig. 4A).

### 5.5 Forward pumping

The flow rate varies depending on the forward speed of the rack gear. Therefore, to adjust the forward speed, different gear ratios must be applied. Changing the gear ratio involves combining gears with different numbers of teeth, resulting in variations in the assembly positions of gears and gear posts. The Table 7 informs the assembly positions of gear posts and the corresponding gears to be assembled based on the gear ratio. By referring to Table 7, assemble R3-1, R3-2, R3-3, and R3-4 in their respective positions 1–4 (marked in the scheme as circled numbers).

### 5.6 **Reverse** pumping

**Fig. 5:**
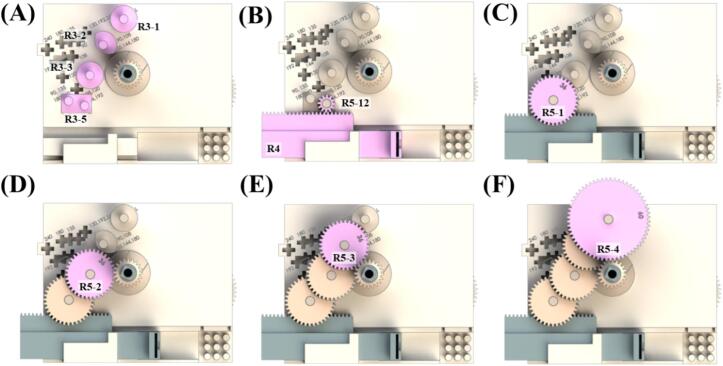
Schematics showing the top layer section assembly orders of reverse pumping.

In this study, to demonstrate a reverse pumping, a gear post was fabricated to enable reverse pumping with the gear ratio is 1:81 as an example. Since Reverse pumping adds a gear that changes the direction of the rack gear, distance from the last gear to the position where the rack gear is fixed should be decreased. Therefore, to achieve different gear ratios, new rack gears need to be designed considering the distance from the last gear to the position where the rack gear is fixed. The assembly process is identical to the forward pumping, except for the substitution of R3-5 for R3-4 and the additional assembly of R5 which is a gear with 12 gear teeth. Fig. 5. illustrates the assembly positions of gear posts and the corresponding gears to be assembled based on the gear ratio.

### 5.7 **Syringe** assembly

**Fig. 6:**
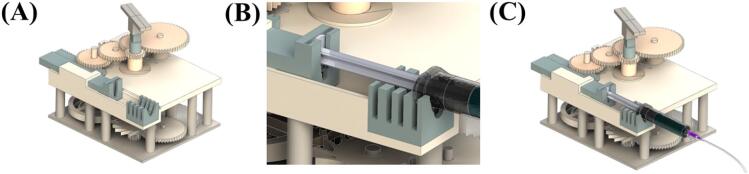
Schematics showing the top layer section assembly orders of reverse pumping.
2) Assemble the syringe plunger onto R4 and syringe barrel onto R6. In this process, assemble the syringe barrel into the gap of R6 based on the length of the syringe plunger (Fig. 6B and 6C).

1) Assemble winding key (R7) onto E3 (Fig. 6A)

### 5.8 Final assembly

**Fig. 7:**
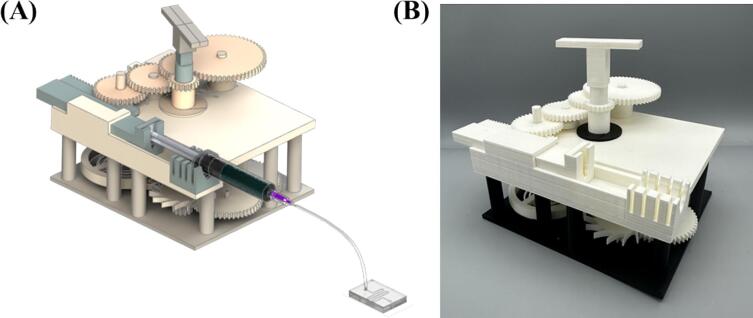
(A) Schematic showing the PRNTD pump prepared for microfluidic operation and (B) fabricated PRNTD pump after assembly.

Fig. 7 shows the assembled model of the PRNTD pump. Video S1 shows the entire assembly procedure of the PRNTD pump.

## 6 Operation instructions

Syringe pump operation instructions
  1) Fix E7 (balance wheel) to prevent movement of the balance wheel while winding up the mainspring. (Placing a small weight, for example, a pen on top effectively hinders its movement).
  2) Rotate the R7 clockwise until M1 is fully wound.
  3) To minimize backlash (the gap between the gears that needs to be closed in order for the track gear to begin pushing the syringe), remove R5-4 and rotate R5-3 counterclockwise (for forward pumping) to allow fluid to be dispensed from the syringe. When the dispensing of the fluid is observed, hold on to R5-3 while reassembling R5-4.
  4) Release the fixation on E7 to allow the operation of the escapement. User may need to give a nudge to E7 to initiate movement.
  5) For preconditioning of a newly printed spring, it is recommended to go through two cycles of operation (two repeats of winding and unwinding of the spring in an assembled pump) [27].
  6) A spring is recommended for a maximum of 30 cycles of use [27].

## 7 Validation and characterization

To characterize the syringe pump, we measured the flow rate. To obtain the flow rate, the dispensed weight from the syringe was measured by an electronic balance (PIONEER®, Ohaus, New Jersey, USA), and then the flow rate was calculated based on the dispensed water over time after converting weight to volume. The characterized flow rate of PRNTD pump is reported in previous work [29].

### 7.1 Characterization of the flow rate

**Fig. 8:**
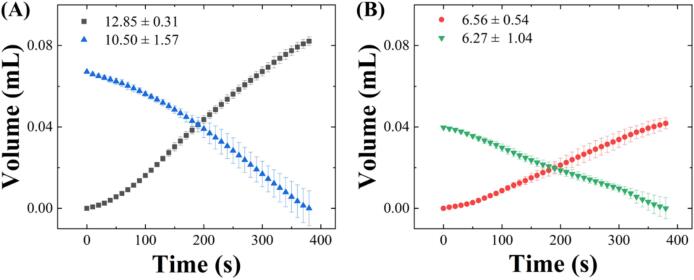
Flow rate characterization of the PRNTD pump. Graph showing the volume of the dispensed water over time of forward pumping and reverse pumping using (A) 5 mL and (B) 3 mL with gear ratio 81. Both experiments were performed in triplicate.

The flow rate is the product of the cross-sectional area of the internal diameter of the syringe barrel and the velocity at which the syringe pump moves the syringe plunger. Therefore, the velocity of the syringe plunger and the size of the syringe affect the flow rate. The variation in flow rate based on syringe size and the velocity of the syringe plunger can be found in the original research paper. In this paper, flow rate of forward pumping and reverse pumping were characterized using different syringe sizes. Experiment was performed three times for each condition. Fig. 8A presents the dispensed volume over time measured using a 5 mL syringe. The theoretical flow rate under these conditions is 16.00 µL/min. The flow rates for forward pumping and reverse pumping were 12.85 ± 0.32 µL/min and 10.50 ± 1.57 µL/min, respectively. The R^2^ values obtained from the linear fitting were 0.9841 and 0.9569. Fig. 8B shows the dispensed volume over time using a 3 mL syringe, with a theoretical flow rate of 9.3 µL/min under these conditions. The flow rates for forward pumping and reverse pumping were 6.56 ± 0.54 µL/min and 6.27 ± 1.04 µL/min, respectively, with linear fitting R^2^ values of 0.9340 and 0.9982. The R^2^ value indicates how close the data is to a linear regression graph, and the closer the R^2^ value is to 1, the more linear the data graph is. Therefore, if the R^2^ value is close to 1, the increment of dispensed volume per time is linear and consistent, which indicates the pumping is maintained without decreasing over time.

Even when 3D printing is performed under consistent conditions, variations may occur due to the moisture content of the filament and the alignment of the 3D printer's axis. Thus, pumps fabricated using different filaments and different 3D printers may result in different flow rates. Additionally, the shape of the mainspring, especially when made from PLA, can deform with multiple usage, affecting pump’s duration. However, a consistent fluid pumping volume is maintained, it can be applicable for microfluidic device operation. Therefore, it is recommended that users test their pump’s flow rate and verify whether the flow rate of the manufactured pump is similar to the optimized syringe pump flow rate before applying it to experiments for microfluidic devices. When using softer materials like ABS, the flow rate may be reduced because the spring provides less torque compared to using PLA, a stiffer material (Fig. S1). The reduced stiffness also results in shorter operation time because the lower torque from the softer spring limits the extent to which the spring unwinds. To allow syringe pump operation with more accurate and precise performance, further design development, such as a calibration function for the flow rate, is required. We expect that further design modifications will allow the widespread use of the PRNTD pump as a pressure source for microfluidic devices. We expect that PRNTD pump will contribute to various fields of study including microfluidic research in resource-limited settings and its applications in POCT and on-site detection.

Funding

This work was supported by a Research Grant of Pukyong National University(2023).

Specifications table

**Table:** 

Hardware name	PRNTD pump
Subject area	• Engineering and materials science Educational tools and open-source alternatives to existing infrastructure
Hardware type	• Biological sample handling and preparationMechanical engineering and materials science
Closest Commercial Analog	Koru Medical Systems FREEDON60 syringe infusion system https://https://www.korumedical.com/products/freedom60
Open-source License	TAPR OHL
Cost of Hardware	$25–30
Source File Repository	https://doi.org/10.17632/28dxsg7x2t.1

### CRediT authorship contribution statement

**Se Been Park:** Writing – review & editing, Writing – original draft, Visualization, Validation, Methodology, Investigation, Formal analysis, Data curation, Conceptualization. **Joong Ho Shin:** Writing – review & editing, Writing – original draft, Supervision, Resources, Project administration, Investigation, Funding acquisition, Formal analysis, Conceptualization.

## Declaration of competing interest

The authors declare that they have no known competing financial interests or personal relationships that could have appeared to influence the work reported in this paper.

## References

[1] Whitesides G.M. (2006). The origins and the future of microfluidics. Nature.

[2] Nguyen N.-T., Wereley S.T., Shaegh S.A.M. (2019). Fundamentals and applications of microfluidics. Artech House.

[3] Altintas Z., Akgun M., Kokturk G., Uludag Y. (2018). A fully automated microfluidic-based electrochemical sensor for real-time bacteria detection. Biosens. Bioelectron..

[4] Pol R., Céspedes F., Gabriel D., Baeza M. (2017). Microfluidic lab-on-a-chip platforms for environmental monitoring. TrAC Trends Anal. Chem..

[5] Park J., Lee K.G., Han D.H., Lee J.-S., Lee S.J., Park J.-K. (2021). Pushbutton-activated microfluidic dropenser for droplet digital PCR. Biosens. Bioelectron..

[6] Eduati F., Utharala R., Madhavan D., Neumann U.P., Longerich T., Cramer T., Saez-Rodriguez J., Merten C.A. (2018). A microfluidics platform for combinatorial drug screening on cancer biopsies. Nat. Commun..

[7] Liu X., Zheng W., Jiang X. (2019). Cell-based assays on microfluidics for drug screening. ACS Sensors.

[8] Kim H.S., Devarenne T.P., Han A. (2015). A high-throughput microfluidic single-cell screening platform capable of selective cell extraction. Lab Chip.

[9] Van Duinen V., Trietsch S.J., Joore J., Vulto P., Hankemeier T. (2015). Microfluidic 3D cell culture: from tools to tissue models. Curr. Opin. Biotechnol..

[10] Jang M., Kim H.N. (2023). From single-to multi-organ-on-a-chip system for studying metabolic diseases. BioChip J..

[11] Pellegrino M., Sciambi A., Treusch S., Durruthy-Durruthy R., Gokhale K., Jacob J., Chen T.X., Geis J.A., Oldham W., Matthews J. (2018). High-throughput single-cell DNA sequencing of acute myeloid leukemia tumors with droplet microfluidics. Genome Res..

[12] Kim T., Jo K. (2023). Microfluidic device to maximize capillary force driven flows for quantitative single-molecule DNA analysis. BioChip J..

[13] Wang Y., Luo S., Kwok H.Y., Pan W., Zhang Y., Zhao X., Leung D.Y. (2021). Microfluidic fuel cells with different types of fuels: A prospective review. Renew. Sustain. Energy Rev..

[14] I. New Era Pump Systems, https://www.syringepump.com/NE-300.php, (accessed.

[15] k. Scientific, https://www.kdscientific.com/legato-200-syringe-pump.html, (accessed.

[16] Pooke F., Payne M., Holder-Pearson L., Heaton D., Campbell J., Chase J.G. (2023). Low-cost, low-power, clockwork syringe pump. HardwareX.

[17] Booeshaghi A.S., Beltrame E.d.V., Bannon D., Gehring J., Pachter L. (2019). Principles of open source bioinstrumentation applied to the poseidon syringe pump system. Sci. Rep..

[18] Wijnen B., Hunt E.J., Anzalone G.C., Pearce J.M. (2014). Open-Source Syringe Pump Library. Plos One.

[19] Amarante L.M., Newport J., Mitchell M., Wilson J., Laubach M. (2019). An open source syringe pump controller for fluid delivery of multiple volumes. Eneuro.

[20] Samokhin A. (2020). Syringe pump created using 3D printing technology and arduino platform. J. Anal. Chem..

[21] Gervasi A., Cardol P., Meyer P.E. (2021). Open-hardware wireless controller and 3D-printed pumps for efficient liquid manipulation. HardwareX.

[22] Kujawa M., Motała S., Gonet M., Pietrzyk R., Czechowski T., Baranowski M. (2021). Low-cost, programmable infusion pump with bolus mode for in-vivo imaging. HardwareX.

[23] Lake J.R., Heyde K.C., Ruder W.C. (2017). Low-cost feedback-controlled syringe pressure pumps for microfluidics applications. PLoS One.

[24] Wu Y., Chen Y., Cheng Y. (2024). Building an Arduino-Based Open-Source Programmable Multichannel Syringe Pump: A Useful Tool for Fluid Delivery in Microfluidics and Flow Chemistry. J. Chem. Educ..

[25] Garcia V.E., Liu J., DeRisi J.L. (2018). Low-cost touchscreen driven programmable dual syringe pump for life science applications. HardwareX.

[26] Czech T.L., Nelson P.P., Thölken C., Meyer P., Hess T., Chung H.-R., Adhikary T. (2024). Pi-seq—A customizable multichannel syringe pump for microfluidics. HardwareX.

[27] Baas S., Saggiomo V. (2021). Ender3 3D printer kit transformed into open, programmable syringe pump set. HardwareX.

[28] Han W., Kim S., Shin S., Yang S.Y., Choi S., Shin J.H. (2021). Wind-up precision pump for portable microfluidics. Sens. Actuators B.

[29] Park S.B., Shin J.H. (2024). Fully 3D-printed, nonelectric, spring-powered syringe pump for operating microfluidic devices. Sens. Actuators B.

