# Supplementary material for: Open-source spring-driven syringe pump with 3D-printed components for microfluidic applications
Source: HardwareX. 2024 Jul 6;19:e00550. doi: 10.1016/j.ohx.2024.e00550 (PMC11299592; doi:10.1016/j.ohx.2024.e00550)
Supplement: Supplementary Data 1 [file mmc1.docx]

**Supplementary Material**

Open-source spring-driven syringe pump with 3D-printed components for microfluidic applications

Se Been Park^a^ and Joong Ho Shin^a,b*^

^a^Industry 4.0 Convergence Bionics Engineering, Pukyong National University

^b^Major of Biomedical Engineering, Division of Smart Healthcare, College of Information Technology and Convergence, Pukyong National University, Busan 48513, Republic of Korea

*To whom correspondence should be address (E-mail: jhshin@pknu.ac.kr)

**Comparison of filament material**

To investigate the effect of material stiffness on mainspring performance, we observed the flow rate by changing the mainspring and escapement components (escapement wheel, balance wheel, and hairspring) to ABS and compared it to those printed with PLA. The parts for additional experiment were printed from a 3D printing company (KLabs, Ulsan, Korea), using PLA filament (TreeD, Italia) and ABS filament (eSUN, China). We measured the dispensed volume versus time using 5 mL syringe, whose theoretical flow rate was set to 16.00 µL/min. The flow rates for PLA parts and ABS parts were 12.52 ± 0.28 µL/min (n=3) and 10.12 ± 0.45 µL/min (n=5). We also measured the dispensed volume over time using 3 mL syringe whose theoretical flow rate was set to 9.3 µL/min. The flow rates for PLA parts and ABS parts were 7.95 ± 0.64 µL/min (n=4) and 5.87 ± 0.18 µL/min (n=4). The two results are consistent in terms of the difference in flow rate and pumping duration. The reduced flow rate of the pump using ABS parts indicates that the lower stiffness provides less torque compared to that of PLA parts. Additionally, the ABS mainspring fails to unwind as much as PLA mainspring, which explains why the pumping ends earlier when the parts are printed with ABS.

The results show that a pump printed using PLA has longer pump operation times and have flow rate closer to the theoretical flow rate than ABS pump. Therefore, as long as the pump is operating under a tolerable stress, the brittleness of PLA does not appear to be a problem and its high stiffness appears to be more suitable than ABS when being used as a printing material for constructing the PRNTD pump.

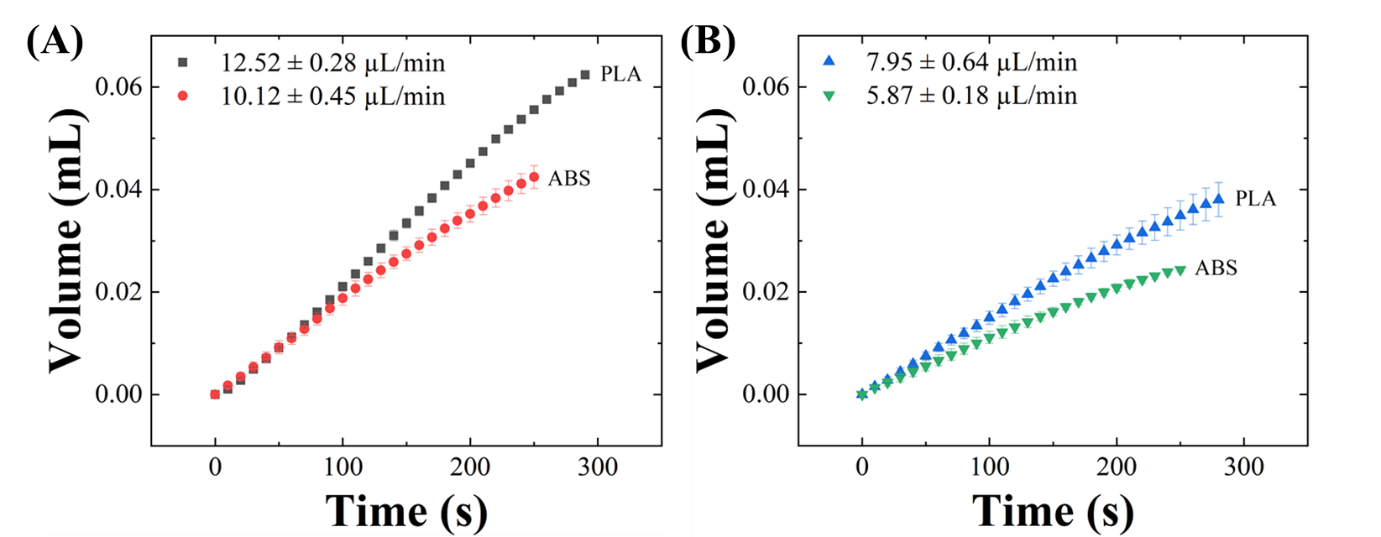


Fig. S1. Graphs showing the measured dispensed volume versus time using PLA pump and ABS pump with the gears set for a theoretical flow rate of (A) 16.00 µL/min and (B) 9.3 µL/min.
